# The Ten Health Commandments

**Published:** 1891-02

**Authors:** 


### The Ten Health Commandments.

1. Thou shalt have no other food than at meal time.

2. Thou shalt not make unto thee any pies, or put into pastry the likeness of any thing that is in the heavens above, or in the earth beneath, or in the waters under the earth. Thou shalt not fall to eating it, or trying to digest it. For the dyspepsia will be visited upon the children to the third and fourth generation of them that eat pie; and long life and vigor upon those that live prudently and keep the laws of health.

3. Remember thy bread to bake it well; for he will not be kept sound that eateth his bread as dough.

4. Thou shalt not indulge sorrow or borrow anxiety in vain.

5. Six days shalt thou wash and keep thyself clean ; and the seventh thou shalt take a great bath, thou, and thy son, and thy daughter, and thy man-servant, and thy maid-servant, and the stranger that is within thy gates. For in six days man sweats and gathers filth and bacteria enough for disease; wherefore the Lord hath blessed the bath-tub and hallowed it.

6. Remember thy sitting-room and bed-chamber to keep them ventilated, that thy days may be long in the land which the Lord thy God giveth thee.

7. Thou shalt not eat hot biscuit.

8. Thou shalt not eat thy meat *fried.*

9. Thou shalt not swallow thy food unchewed, or highly spiced, or just before hard work, or just after it.

10. Thou shalt not keep late hours in thy neighbor's house, nor with thy neighbor's wife, nor his man-servant, nor his maid-servant, nor his cards, nor his glass, nor with any thing that is thy neighbor's—Austin Bierbower, in *Phrenological Journal.*

---

Uniform Nomenclature in Anatomy.—The establishment of a uniform nomenclature in anatomy, which was taken in hand by German anatomists about a year ago, has now become an international affair, and the committee appointed for the purpose, which has hitherto consisted exclusively of Germans, now numbers three foreign members—namely, Leboucq, of Geneva; Cunningham, of Edinburgh; and Romiti, of Pisa. The expenses of the task are to be borne by the learned corporations of Germany, because the Anatomical Society, which began it, does not possess the necessary funds. The Prussian, Bavarian, and Saxon academies of science have contributed 1,500 marks (nearly £75) each, the Academy of Vienna, 1,000 guildens (about £85), and the Anatomical Society 1,000 marks (nearly £50). The completion of the work will be entrusted to a commission presided over by Professor von Kolliker, of Wurzburg. The preliminary work is to be done by an anatomist of special qualifications, including the necessary philological attainments. —*Lancet.*

